# Evaluation of Onco E6 point of care rapid diagnostic test for human papilloma virus in Bahir Dar, Amhara Regional State, Ethiopia

**DOI:** 10.1371/journal.pone.0321076

**Published:** 2025-04-16

**Authors:** Alemayehu Abate, Abaineh Munshea, Endalkachew Nibret, Belay Bezabih, Bereket Amare, Gizachew Yismaw

**Affiliations:** 1 Health Biotechnology Division, Institute of Biotechnology, Bahir Dar University, Bahir Dar PO, Ethiopia; 2 Amhara Public Health Institute, Bahir Dar PO, Ethiopia; 3 Biology Department, College of Science, Bahir Dar University, Bahir Dar PO, Ethiopia; 4 Department of Pathology, Afilas General Hospital, Bahir Dar, Ethiopia; Teikyo University, School of Medicine, Japan

## Abstract

**Introduction:**

Cervical cancer is a malignant tumor arising from the cells of the uterine cervix. The oncogenic human papillomavirus (HPV) infection is the main causative agent of cervical cancer. Effective HPV screening program can lead to a significant reduction in the morbidity and mortality associated with this cancer.

**Objective:**

The aim of the study was to evaluate the diagnostic performance of OncoE6™ cervical test kit for cervical precancer and cancer in Amhara Regional State, northwest Ethiopia.

**Methods:**

An institute-based cross-sectional study was conducted at Felege Hiwot Compressive Specialized Hospital which is found in Bahir Dar, Amhara Regional state. A total of 297 samples were collected. A sterile, disposable speculum was inserted, without lubricant, and two swabs were taken using the “Tipped Polyester” (Dacron) swab provided with the Onco *E6*™ kit. Swabs taken were tested for onco E6 proteins testing as per the manufacturer’s protocol and pap smear for cytology test. A punch of biopsy was also taken for histopathological diagnosis. Data for all samples were collected using pre-prepared excel database for onco E6 test, pap cytology and punch histopathology. All the data were coded and entered into Epi-info and transported to SPSS version 26.0 software package for analysis.

**Results:**

Out of 56 (18.86%) participants who tested positive on the histo-pathological diagnosis, Onco E6 was positive in 32 (57.14%), negative in 24 (42.85%). Of 241 participants who tested negative on the histo-pathological diagnosis, Onco E6 was positive in 5 (2.07%) and negative in 236 (97.9%). OncoE6™ cervical test kit had a sensitivity of 57.14% (95% CI: 43.22%–70.29%) and specificity of 98% (95% CI: 95.23%–99.32%) with positive predictive value of 86.6% (95% CI: 71.36%–95.53%), negative predictive value of 90.76% (95% CI: 86.49%–93.93%), and accuracy of 90.18% (95% CI: 86.21%–93.31%).

**Conclusion:**

The HPV 16/18 OncoE6™ Cervical test kit test had sub-optimum sensitivity and high specificity for detection of cervical precancer and cancer cases. The sensitivity of the kit could be increased by incorporating other more prevalent genotypes like genotype 52, 58, 31 and 35. The HPV16/18-E6 test could be used either as primary screening tool or in conjunction with other diagnostic methods.

## Introduction

Cervical cancer is a malignant tumor that develops from the uterine cervix cells. The primary cause of cervical cancer is the oncogenic human papillomavirus (HPV) infection [1]. Around 70% of cervical cancer cases globally are caused by the two most significant high-risk HPV strains, HPV-16 and 18 [2]. Worldwide, there are around 604,127 new cases of cervical cancer diagnosed each year (estimates for 2020). Cervical cancer is the fourth most common cause of cancer in women worldwide, and the second most frequent cancer in women worldwide between the ages of 15 and 44 [3]. In developing nations, cervical cancer is second to breast cancer in terms of both incidence and death [4].Although it may be prevented to a great extent, 36 low- and middle-income countries (LMICs) still have it as the leading cause of cancer-related deaths among women [5].

In Ethiopia, cervical cancer is the most frequent malignancy among women between the ages of 15 and 44; it is a public health problem [6]. According to estimates from 2022, 5,338 women lose their lives to cervical cancer each year, while 7,445 new cases are identified with the disease annually [7].

There is a considerable gap in cervical cancer diagnosis, particularly in resource challenged settings. Majority of resource-limited nations have not been able to effectively execute such screening programs due to numerous reasons such as lack of suitable infrastructure, qualified workforce and absence of political will [8]. Point-of-care (POC) testing that particularly identifies transforming HPV infections may increase the accuracy of cervical cancer screening programs [9].

The World Health Organization states that POC tests that address the need for disease control, particularly in developing nations, must fulfill the following “ASSURED” requirements: Affordable, sensitive, specific, fast, robust, equipment-free, user-friendly, robust, affordable, and deliverable to end users are the first seven criteria [10]. There is a substantial gap in cervical cancer diagnosis, particularly in resource limited settings due to luck of good performing diagnostic modality, low accessibility of cervical cancer testing, inconvenient testing hours.

A novel technique to diagnose clinical samples for the presence of HPV E6and E7 oncoproteins of high risk genotypes is manufactured in Bahir Dar, Ethiopia. The prognostic value of this method and its possible application as diagnostic and predictor of cervical cancer progression give a better choice for prevention and management of cervical cancer. The objective of this study was to evaluate the performance of the Onco E6 HPV test relative to the cytology and histopathology examinations.

## Materials and methods

### Study setting and design

An institute-based cross-sectional study was conducted at Felege Hiwot Comprehensive Specialized hospital which is found in Bahir Dar, Amhara Regional State from September 20 December 2023. A total of 324 women of age above 30 who visited FHCSH Gynecological unit who came for gynecological complaints were enrolled in the study.

### Study population

The study population was all women presenting with gynecological complaints, age above 30 and HIV positive women with all age range who visited FHCSH Gynecological unit.

### Inclusion and exclusion criteria

Women who came for gynecological complaints such as vaginal bleeding, vaginal discharge, abdominal pain, back pain, difficulty of urination, difficulty of defecation, protruded mass per vagina, burning sensation and itching, pain during sexual intercourse and urination during data collection time were enrolled in the study. However, 27 of them had insufficient PAP smears and they were excluded from the study. The rest 297 were included for the study. All the 297 women gave their informed consent to participate in the study. All women who visited FHCSH gynecological unit, above 30 years old, HIV positive women with all age range and willing to participate in the study were included. All women who took any kind of treatment for cervical cancer or any vaginal medication, vaginal contraceptives or douches 48 hours prior to the test, those who had sexual intercourse 24 hours before the test; women who are in menstruation, pregnant and women who had hysterectomy were excluded from the study.

All of the study participants were screened with Visual acetic acid test (VIA), genotyping of the HPV DNA test and cervical cytology examination with the PAP test. Study participants with positive results from screening tests were examined with colposcopy and biopsy was taken for histopathology.

### Sample size determination

Sample size was determined from formulation of sensitivity and specificity test using Power Analysis and Sample Size (PASS) software based on desired type I error, power and effect size [11]. The minimum sample size was determined by taking the prevalence of a disease 19%, by assuming sensitivity of the kit is comparable with the gold standard [12], and specificity of the kit is greater than 70%, the power is set to be at least 80% and the p-value, is set to be less than 0.05. The sample size for sensitivity and specificity of Onco E6 performance study was 49 positive for histopathology, 196 negative samples with the gold standard histopathology examination and 49 negative controls. By taking 10% contingency, the sample size was 324.

### Sampling technique and procedure

The sampling technique was systematic random sampling. Every third study participants was selected after a random starting study participant who was selected by lottery method.For each participant, Histopathology, cytology using PAP and E6 onco-protein test using Onco*E6*™ Cervical Test kit (Arbor Vita Corporation, Fremont, CA, USA) were performed. A sterile, disposable speculum was inserted, without lubricant, and two swabs were taken using the “Tipped Polyester” (Dacron) swab provided with the Onco*E6*™ kit. Swabs taken tests for onco proteins testing and smear for cytology pap test. A punch of biopsy was also taken for histopathology. All PAP smears and punch biopsies were evaluated without knowledge of the Onco E6 results by two independent experienced pathologists.

### Onco E6 test

The Onco E6 cervical test is an immunochromatographic test using lateral flow format. Three test strips constitute one test unit of type E6-16 and E6-18, with each test strip allowing for analysis of one individual clinical specimen and several units (of 3 test strips each) can be used in parallel by one operator. A control line is included on each strip, which allows for verification of detector reagent activity and proper sample solution migration up the test strip. The time from sample collection to test results is typically approximately 2.5 hours. Briefly, a cervical specimen collected using a polyester swab was stored in a tube without buffer until tested. The specimen was prepared sequentially by treating the swab with a lysis solution (15 minutes), a condition solution (15 seconds), and then clarifying the specimen solution using a table-top microcentrifuge.

### PAP test

A pap smear, also called a pap test is an examination to detect changes in the cervical cells. It takes 10–20 minutes for the whole examination of stained slides. To collect the sample a speculum was inserted in to the vagina to hold the wall of the vagina which helps to see the cervix, and then a soft brush was inserted to scrap the cervix to collect the sample and which was then smeared to the slide glass. The slide smear was stained using methanol, hematoxyline, and eosin and distilled water. The stained smear was examined under a microscope for cellular abnormalities and classifies the result as normal, ASC-US, LSIL, HSIL and invasive carcinoma.

### Histopathology test

Histological examination of cervical biopsy samples is deemed the gold standard for the diagnosis of pre cancer lesions and cervical cancer (CaCx) and it is used to clarify the staging of CIN. To take the punch biopsy, the cervix was inspected at low-power magnification (5x to 10x), to look for areas of abnormality and identify the transformation zone and the original and new squamocolumnar junctions. After preparation, the punch biopsy was collected for examining cellular abnormalities and classify the result as normal, atypia, CIN I, CIN II, CIN III and invasive carcinoma.

### Ethical consideration

The study was approved by the Institutional Review Board of Amhara Public Health Institute. All participants provided written informed consent, and all of the identifying information for the patients was maintained coded in a database to ensure data confidentiality and privacy of the participants. Women who screened positive for HPV were checked for cervical dysplasia and treated as appropriate.

### Statistical analysis

All the data were coded and entered into Epi-info and transported to SPSS version 26.0 software package for analysis. For controlling errors 10% of the data were double entered, also frequency checks were done. The data were analyzed using the accuracy measures used (sensitivity, specificity, positive predictive value [PPV], and negative predictive value [NPV]) to determine the performance characteristics of Onco E6 cervical cancer diagnosis kit. The result of performance evaluation was presented in a two by two table taking Onco E6 as a test method and Punch histopathology result as a Gold Standard.

## Results

### Demographic and clinical characteristics

The study included a total of 297 women aged above30 years with median age of 49 years to evaluate the diagnostic performance of Onco E6 protein test kit. About 61% of the participants lived in rural area, more than 84% of them were illiterates and 63% of them were married. Demographic and clinical characteristics of the participants are presented in Table 1.

**Table 1:** Demographic and clinical characteristics of the study participants attending cervical cancer screening unit in FHCSH.

Variables	Categories	Frequency	Percent
Age (year)	<30	28	9.4
	30–50	185	62.3
	>50	84	28.3
Educational status	Illiterate	251	84.5
	Read and write	15	5.1
	Elementary	11	3.7
	High school and above	20	6.7
Monthly income	<2000 birr	62	20.9
	2001-5000 birr	108	36.4
	>5000 birr	127	42.8
Marital status	Single	44	14.8
	Married	187	63.0
	Divorced	27	9.1
	Widowed	39	13.1
Occupation	Employed	108	36.4
	Others	189	63.6
Residence	Rural	182	61.3
	Urban	115	38.7
HIV status	Positive	59	20
	Negative	238	80

### Histo-pathological and Pap test results

Histo-pathological diagnosis had confirmed test results as negative for Dysplasia/malignancy, CIN I, CIN II, CIN III, and cervical cancer cases. From the total number of participants, 56 (19%) of them had histo-pathological positive results. Pap test results were reported as negative dysplasia/malignancy, low-grade squamous intraepithelial lesion (LSIL), High-grade squamous intraepithelial lesion (HSIL) and carcinoma. From the total number of participants, 225 (75.8%), 19 (6.4%), 23 (7.7%) and 30 (10.1%) were negative for dysplasia/malignancy, low-grade squamous intraepithelial lesion (LSIL), High-grade squamous intraepithelial lesion (HSIL) and carcinoma respectively (Table 2).

**Table 2:** Pap test results compared to histopathological results of the study participants attending cervical cancer screening unit in FHCSH.

Histopathology Result					
	CIN II+		CIN II ^–^		Total
Pap test result	Onco E6 +	Onco E6 -	Onco E6 +	Onco E6 -	
Negative for dysplasia	3	3	3	216	225
LSIL	1	2	2	14	19
HSIL	13	6	0	4	23
Carcinoma	15	13	0	2	30

### Onco E6 protein test results

The OncoE6™ cervical test kit used in this study is specific for HPV16 and HPV18 E6 onco-protein. The result of Onco E6 test indicated that 37 (12.46%) of study participants had positive for HPV type 16 and 18 and 260 (87.54%) had negative results. Among HPV E6 oncoprotein positive cases, 32 (86.5%), 3 (8.1%), 2(5.4%) were positive for type 16, type 18 and both type 16 and 18 respectively.

### Performance evaluation of Onco E6 test kit

All of the study participants underwent Onco E6 test, Pap test and histo-pathological test. The result of histopathology examination indicated that 56 (18.86%) of the study participants were diseased and 241 (81.14%) were not diseased. Out of 56 (18.86%) participants who tested positive on the histo-pathological diagnosis, Onco E6 was positive in 32 (57.14%), negative in 24 (42.85%). Of 241 participants who tested negative on the histo-pathological diagnosis, Onco E6 was positive in 5 (2.07%) and negative in 236 (97.9%).

The performance of the OncoE6™ Cervical test kit was evaluated by sensitivity, specificity, PPV and NPV. The sensitivity, specificity, positive predictive value and negative predictive value were calculated taking OncoE6™ Cervical test kit as test method and Hiosto-pathological test as gold standard method. The result is presented in a two by two table (Table 3).

**Table 3:** Diagnostic performance of OncoE6™ Cervical test kit for cervical pre-cancer and cancer of the study participants attending cervical cancer screening unit in FHCSH.

Histo-pathological test result						
**OncoE6™ Cervical test kit results**		**Positive**	**N**	**Negative**	**N**	**Total**
	**Positive**	True Positive	a= 32	False Positive	c= 5	a + c = **37**
	**Negative**	False Negative	b= 24	True Negative	d=236	b + d = **260**
	**Total**		a + b = **56**		c + d = **241**	

The performance of OncoE6™ Cervical test kit is expressed by sensitivity 57.14% (95% CI: 43.22%–70.29%), specificity 98% (95% CI: 95.23%–99.32%), PPV 86.6% (95% CI: 71.36%–95.53%), NPV 90.76% (95% CI: 86.49%–93.93%), and Accuracy 90.18% (95% CI: 86.21%–93.31%) (Table 4).

**Table 4:** Performance characteristics of Onco E6 test compared with histopathology results among women in Bahir Dar, northwest Ethiopia.

Methods	Sensitivity (95% CI)	Specificity (95% CI)	PPV (95% CI)	NPV (95% CI)	Kappa Value
Onco E6 test	57.14% (43.22–70.29)	98% (95.23–99.32)	86.6% (72.50–94.06)	90.76% (87.80–92.95)	0.633

## Discussion

Human papillomavirus E6/E7 oncoproteins are overexpressed after HPV invasion into the host cervical cells in the form of episomal HPV DNA or viral integration into the host’s genome, and are closely and directly related to the development of cancers. The use of rapid diagnostic tests for HPV infection and production of onco protein can greatly enhance screening, confirm clinical case and monitor prognosis of cervical cancer, especially in resource poor settings. In the present study we have evaluated a commercially available Onco E6 HPV type 16 and 18 point of care test kit manufactured in Medx Diagnostics PLC, Bahir Dar, Ethiopia in Joint Venture with Arbor Vita Corporation, USA.

The results of the current study showed thatHPV16/18 E6 oncoprotein test has optimum sensitivity and high specificity which agrees with findings from other study conducted in Bangladesh which states the sensitivity, specificity, PPV, and NPV of the E6 onco-protein test were 52% (95% CI: 31, 73), 97% (95% CI: 92, 99), 75% (95% CI: 47, 92), and 92% (95% CI: 86, 96) [13].

The results our study are almost similar to findings in China in a study conducted among 7,543 women, aged 25–65, who resided in rural China, that indicated the sensitivity of the OncoE6 test for CIN2^+^ and CIN3^+^ were 42.4% and 53.5%, respectively. OncoE6, on the other hand, was 99% specific for CIN2^+^ and CIN3^+^ [14].

The current study is also similar with a study done in Brazil which showed that HPV16/18-E6 detection showed a low level of sensitivity for CIN2+ or CIN3+ detection, but it was highly specific for HPV16/18-E6 detection: sensitivity 49.6 (95% CI: 40.3–58.9) and specificity 91.8 (95% CI: 88.3–94.5) [15].

Our result has a similar finding with a study conducted in Honduras which states the OncoE6 test was found to have a predicted sensitivity of 56.4 (95% CI: 43.3–68.6) and specificity of 97.5% (95% CI: 93.7–99.0), respectively [16].

The optimum sensitivity of Onco E6 test kit for detection of precancer and cancer is because the kit can detect only two prevalent HPV types (Type 16 and 18). This result is consistent with a study conducted in China indicated that findings of the OncoE6 cervical test showed a high positive predictive value (PPV) but lower sensitivity [17].This is a direct reflection of the prevalence and distribution of HPV genotypes circulated in Ethiopia. The restriction of the test to HPV 16/18 E6 oncoprotein detection constitutes an inherent limitation of the method that can be improved with the addition of other high prevalent HPV types such as genotype 52, 58, 31 and 35, which are more prevalent in the study area [18]. This was supported by a study done in China that suggest adding HPV types other than type 16/18 to the assay’s would increase targeting of HPV types in order to improve the OncoE6 cervical Test’s applicability[19].

Our study identified that Onco E6 test has high specificity, PPV and NPV. The high specificity and high positive and negative predictive value of the OncoE6 test kit for cervical precancer and cancer makes the kit a good choice to be used as a screening test particularly in regions with high HPV-prevalence and high-risk populations, such as HIV and other STI. Our result is similar with a study done in china that indicated because of its high specificity, it can be used to screen high-risk, high-HPV-prevalence populations, such as HIV-positive women, for cervical cancer [20].This result is similar with a study done in Bangladish which states that by using the OncoE6 test for primary screening, a sensitivity comparable to or higher than that of the widely-used VIA test may be attained. However, due to its significantly higher specificity than VIA screening, HPV E6 screening may lessen overtreatment in the context of a screen and treat program [21]. Our result is also consistent with a study in China which states using the E6 oncoprotein during screening could significantly lower the number of unnecessary referrals for colposcopy because it is a highly specific biomarker in HPV positive triage[22,23].

### Limitation of the study

There are limitations in this study. The performance of the Onco E6 kit was not fully explored in HIV and other STI positive women, which could affect its sensitivity and specificity. The other limitation was study populations were women with gynecological complaints, which could be difficult to generalize the findings to women who does not show any symptom.

## Conclusion and recommendation

The OncoE6™ Cervical test kit detects E6 oncoproteins of Human papilloma virus type 16 and 18 from cervical swabs, using monoclonal antibodies (MAbs) in a lateral-flow strip format. The HPV 16/18 OncoE6™ Cervical test kit test had sub-optimum sensitivity and high specificity for detection of cervical precancer and cancer cases. The sensitivity of the kit could be increased by incorporating other more prevalent genotypes like genotype 52, 58, 31 and 35. It could fill the diagnostic gap in cervical cancer diagnosis, particularly in resource limited settings due to the lack of good performing diagnostic modality, low accessibility of cervical cancer testing. The HPV16/18-E6 test could be used either as primary screening tool or in conjunction with other diagnostic methods like VIA, HPV DNA test and PAP test, and could be used as an effective triage test to reduce colposcopy referrals.
